# P38 MAPK Promotes Migration and Metastatic Activity of BRAF Mutant Melanoma Cells by Inducing Degradation of PMCA4b

**DOI:** 10.3390/cells9051209

**Published:** 2020-05-13

**Authors:** Randa Naffa, Lisa Vogel, Luca Hegedűs, Katalin Pászty, Sarolta Tóth, Kornélia Kelemen, Neha Singh, Attila Reményi, Enikő Kállay, Mihály Cserepes, József Tóvári, Michael Grusch, Ágnes Enyedi

**Affiliations:** 1 2nd Institute of Pathology, Semmelweis University, H-1091 Budapest, Hungary; rnaffa1410@yahoo.com (R.N.); kornelia.kelemen@turbine.ai (K.K.); 2 Institute of Cancer Research, Department of Medicine I, Medical University of Vienna, Vienna A-1090, Austria; lisa.wimmer91@gmail.com (L.V.); michael.grusch@meduniwien.ac.at (M.G.); 3 Department of Thoracic Surgery, Ruhrlandklinik, University Clinic, D-45239 Essen, Germany; luca.hegedues@rlk.uk-essen.de; 4 Department of Biophysics and Radiation Biology, Semmelweis University, H-1094 Budapest, Hungary; csokay.katalin@med.semmelweis-univ.hu; 5 Department of Anatomy, Cell and Developmental Biology, Eötvös Loránd University, H-1117 Budapest, Hungary; szipp07@gmail.com; 6 Institute of Organic Chemistry, Research Centre for Natural Sciences, HAS, H-1117 Budapest, Hungary; neha.singh@ttk.hu (N.S.); remenyi.attila@ttk.mta.hu (A.R.); 7 Institute of Pathophysiology and Allergy Research, Center for Pathophysiology, Infectiology and Immunology, Medical University of Vienna, Vienna A-1090, Austria; enikoe.kallay@meduniwien.ac.at; 8 National Institute of Oncology, Department of Experimental Pharmacology, H-1122 Budapest, Hungary; cserepestm@gmail.com (M.C.); tozsi@oncol.hu (J.T.)

**Keywords:** plasma membrane Ca^2+^ATPase isoform 4b, p38 mitogen-activated protein kinase (MAPK), endocytosis, calcium signaling, BRAF mutant melanoma, metastasis

## Abstract

Metastatic melanoma is the most aggressive type of skin cancer. Previously, we identified the plasma membrane Ca^2+^ pump isoform 4b (PMCA4b or *ATP2B4*) as a putative metastasis suppressor in BRAF mutant melanoma cells. Metastasis suppressors are often downregulated in cancer, therefore, it is important to identify the pathways involved in their degradation. Here, we studied the role of p38 MAPK in PMCA4b degradation and its effect on melanoma metastasis. We found that activation of p38 MAPK induces internalization and subsequent degradation of PMCA4b through the endo/lysosomal system that contributes to the low PMCA4b steady-state protein level of BRAF mutant melanoma cells. Moreover, BRAF wild type cell models including a doxycycline-inducible HEK cell system revealed that p38 MAPK is a universal modulator of PMCA4b endocytosis. Inhibition of the p38 MAPK pathway markedly reduced migration, colony formation and metastatic activity of BRAF mutant cells in vitro partially through an increase in PMCA4b and a decrease in β4 integrin abundance. In conclusion, our data suggest that the p38 MAPK pathway plays a key role in PMCA4b degradation and inhibition of this pathway—by increasing the stability of PMCA4b—may provide a potential therapeutic target for inhibition of melanoma progression and metastasis.

## 1. Introduction

Melanoma is an aggressive type of skin cancer that develops from the pigment melanin producing melanocytes. The main cause of 65–90% of all melanomas is the exposure to ultraviolet light (UVR) resulting in DNA damage and accumulation of genetic mutations that eventually lead to cancer [1]. A mutation in the kinase BRAF is observed in approximately 50% of melanomas. Most of these mutations are at codon 600, and over 90% of them are point mutations resulting in a glutamic acid to valine substitution (BRAF^V600E^) [2]. This mutation causes constitutive activation of the BRAF/MEK/ERK MAPK pathway inducing cell growth, survival, invasion and metastasis [3]. Moreover, other signalling pathways could also be affected by BRAF mutation. An interplay between members of the MAPK pathways—ERK, p38 MAPK and c-Jun N-terminal kinase (JNK)—and NFκB in melanoma progression and therapy resistance has been reported [4,5,6].

Calcium plays an important role in many different processes, and any changes in its intracellular homeostasis can affect signaling pathways involved in tumor development and metastasis [7]. A “molecular toolkit” is needed to guarantee proper Ca^2+^ handling necessary to maintain normal cellular Ca^2+^ homeostasis. This toolkit includes Ca^2+^ channels that allow Ca^2+^ to enter the cells and Ca^2+^ pumps (SERCAs and PMCAs) accompanied with the Na^+^/Ca^2+^ exchanger (NCX) in certain cell types for removing excess Ca^2+^ from the cytosol [8]. Any alterations in the expression, regulation and activity of these Ca^2+^ channels and/or Ca^2+^ pumps residing both in the plasma membrane and intracellular organelles result in altered Ca^2+^ homeostasis that may lead to aberrant cell-cycle progression, uncontrolled proliferation and enhanced cell migration, hallmarks of tumorigenesis and metastasis [9,10]. Plasma membrane Ca^2+^ transporting ATPases (PMCAs) are key Ca^2+^ pumps that maintain low levels of Ca^2+^ concentration inside living cells of higher organisms, therefore, they can regulate important cellular processes such as cell cycle progression, apoptosis, or migration [11,12]. There are four genes (*ATP2B1-4*) coding for the four isoforms of PMCA and more than 20 different splice variants are generated by alternative splicing of the primary transcripts [13]. The expression of a particular isoform, PMCA4b (*ATP2B4*), is downregulated in cancer cells from various tumor types, and hence suggested to play a key role during malignant transformation [12,14,15].

In melanomas, changes in the expression of several Ca^2+^ channels/pumps, and consequently alternations in Ca^2+^ signaling have been described in the literature [16]. Previously, our laboratory identified PMCA4b as a putative metastasis suppressor in BRAF mutant melanoma cells where PMCA4b expression is down-regulated [17]. We demonstrated that the Ras-BRAF-MEK-ERK pathway is involved in PMCA4b downregulation and that the BRAF inhibitor vemurafenib increased PMCA4b expression at both the mRNA and protein levels. We also showed that PMCA4b overexpression inhibited migration in vitro and metastatic activity in vivo of BRAF mutant melanoma cells. In addition, we and others demonstrated that besides the BRAF/MEK/ERK pathway the expression of PMCA4b is also modulated by estradiol in ER+ MCF-7 breast cancer cells [18] and histone acetylation in breast [19] and colon [20,21] cancers and in melanoma [22] suggesting that the expression of this pump is under tight control.

During tumor development, metastasis suppressors are often downregulated, thus finding ways for restoring their expression has vital importance. The main goal of the present study was to find new treatment options to restore PMCA4b expression and/or function with possible impact on the metastatic activity of BRAF mutant melanoma cells. We found that in addition to mutant BRAF an increased p38 MAPK activity was responsible for the downregulation of PMCA4b in these cells by decreasing the stability of the PMCA4b protein. The present study demonstrates that inhibitors of the p38 pathway can restore PMCA4b function and reduce migration and metastatic activity of mutant BRAF melanoma cells nearly as efficiently as inhibitors of the BRAF/MEK/ERK pathway, offering a new (additional) mechanism in counteracting (overcoming) resistance to BRAF inhibitors.

## 2. Materials and Methods

### 2.1. Human Cell Lines

#### 2.1.1. Cell Culture

The cell lines under investigation were BRAF (V600E) mutant melanoma cells (A375 and SK-MEL-28), BRAF/NRAS wild-type melanoma cells (MEWO), stably transfected A375 cells (A375-GFP-PMCA4b), stably transfected HeLa cervix adenocarcinoma cells (Hela-GFP-PMCA4b) and stably transfected human embryonic kidney cells (HEK-mCherry-MKK6EE-Dox). HEK-mCherry-MKK6EE-Dox was kindly provided by the Reményi laboratory (MTA-TTK, Budapest, Hungary). MEWO and A375 cells are from ATCC and were subjected to STR analysis at the Medical University of Vienna including the genetically modified A375 cell lines. HeLa cells were purchased from ECACC. The cell lines were cultured in Dulbecco’s modified Eagle’s medium (DMEM) (Lonza, Walkersville, MD, USA) supplemented with 10% fetal bovine serum (FBS) (Thermo Fisher Scientific, Waltham, MA, USA), 1% penicillin-streptomycin (Lonza) and 2 mM L-glutamine (Lonza) maintained at 37 °C in a humidified 5% CO_2_ incubator.

#### 2.1.2. Cell Treatments, Chemical Reagents

Cells were treated with the BRAF^V600E^ inhibitor vemurafenib (PLX4032), JNK inhibitor (SP600125), NF-KB inhibitor (BAY11-7082) and p38 MAPK inhibitors (SB203580, SB202190). All the above inhibitors were purchased from Selleck Chemicals (Munich, Germany), dissolved in DMSO and stored at −80 °C as a stock solution. The proteasome inhibitor (MG132), anisomycin and calcium ionophore A23187 were dissolved in DMSO. Doxycycline hydrochloride (Dox) and chloroquine (CQ) were dissolved in pure water. These reagents were purchased from Sigma-Aldrich (St. Louis, MO, USA) and stored at −20 °C. The final DMSO concentration in the experiment did not exceed 0.01%.

For western blot analysis, cells were seeded at 1.5–2 × 10^5^ cells/well in 6-well plates. For immunofluorescence staining and Ca^2+^ signal measurements, cells were seeded at 1–3 × 10^4^ cells/well in 8-well Nunc Lab-Tek II chambered coverglass (Nalge Nunc International, New York, NY, USA). After a 24-h incubation period, fresh medium was added together with the appropriate drug and incubations continued as indicated.

#### 2.1.3. Generation of Stable Cell Lines

A375-GFP-PMCA4b and Hela-GFP-PMCA4b stable cell lines were generated by transfection of cells with a SB-CAG-GFP-PMCA4b-CAG-Puromycin construct which contains a Sleeping Beauty transposon system as previously described [17]. For calcium measurement studies, the A375-GCAMP6 stable cell line was generated by transfection with a SB-GCAMP6 construct kindly provided by Prof. Orban laboratory (MTA-TTK) as previously described [23].

For making the mCherry-MKK6EE cell line for the specific activation of p38, HEK293T cells were transfected with a pEBDTet vector containing the constitutively activated version of the mCherry-MKK6 fusion construct with phospho-mimicking activation loop residues (MKK6EE) [24]. The stable cell line was established by keeping the cells under puromycine for more than a week, then expression of FLAG-tagged MKK6EE and concomitant specific MAPK activation were monitored by Western-blots after doxycycline (Dox) treatment (2 µg/mL) in DMEM containing 10% FBS. Selective activation of p38 but not that of ERK1/2, ERK5 or JNK was confirmed with western-blots using MAPK phosphorylation state specific antibodies.

#### 2.1.4. Transient Cell Transfection

HEK-mCherry-MKK6EE-Dox inducible cells were seeded in 8-well Lab-Tek II chambered coverglass (Nunc) and allowed to adhere overnight. Cells were transiently transfected with the SB-CAG-GFP-PMCA4b-CAG-Puro plasmid using the FuGENE HD transfection reagent (Promega, Madison, WI, USA) according to the manufacturer’s recommendations. After 24 h media were changed and the appropriate drugs at the appropriate concentration and time were added.

### 2.2. Cell Treatments and Measurements

#### 2.2.1. Quantitative Real-Time Polymerase Chain Reaction

For quantitation of PMCA4b transcripts in both A375 and MEWO cell lines, total RNA was extracted from control and p38 inhibitor (SB202190)-treated cells at concentrations 1, 3, 6, 10, 30 µM using Trizol reagent (Life Technologies, Van Alben Way, CA, USA) according to manufacturer’s recommendations. cDNA synthesis and quantitative PCR-TaqMan assay were performed as described previously [17]. For cDNA synthesis, RevertAid Reverse Transcriptase kit (Thermo Scientific) and for quantification of the transcripts the TaqMan assays Hs00608066_m1 (PMCA4b) and Hs99999905_m1 (GAPDH) (Thermo Scientific) were used.

#### 2.2.2. Western Blot Analysis

Total proteins were extracted from cells by precipitation with 6% TCA. Samples were resolved by electrophoresis in 10% acrylamide gel and then electroblotted onto PVDF membranes (BioRad, Hercules, CA, USA) as described previously [22].

Blots were incubated with the following primary antibodies; Mouse monoclonal antibodies: anti-PMCA4b (JA3, 1:1000, cat. # MABN1801, Sigma-Aldrich), anti-PMCA4 (JA9, 1:1000, cat. # P1494, Sigma-Aldrich), anti-HSP (1:1000, cat. # 2402, Cell Signaling Technology, Danvers, MA, USA), anti-PP38 (1:1000, cat. # 9216, Cell Signaling Technology). Rabbit monoclonal anitbodies (Cell Signaling Technology): anti-phospho-p44/ 42 MAPK (ERK1/2) (1:2000, cat. # 4370), anti-ERK1/2 (MK1) (1:1000, cat. # 9102), anti-PHSP27 (1:1000, cat. # 9709), anti-P38 (1:1000, cat. # 9212), anti-β4-integrin (D8P6C)XP (1:1000, cat. # 14803). Rabbit polyclonal antibodies: anti-β-tubulin (1:1000, Abcam, cat. # ab6046). Detection was achieved using horseradish peroxidase-conjugated anti-rabbit or anti-mouse secondary antibodies (dilution 1:10,000, Jackson ImmunoResearch, West Grove, PA, USA) and visualized with Pierce ECL Western Blotting Substrate (Thermo Scientific). In order to measure protein expression levels, densitometry analysis of specific bands was performed using the ImageJ software v1.42q (National Institutes of Health, Bethesda, MD, USA).

#### 2.2.3. Immunofluorescence Microscopy

A375 or A375-GFP-PMCA4b melanoma cells were cultured in 8-well Lab-Tek II chambered coverglass (Nunc) and allowed to adhere overnight. Next day, cells were treated as indicated in the figure legends. Treatments used in this study included the following concentrations: (0.5 µM vemurafenib, 10 µM p38 inhibitor (SB202190) or 10 µM chloroquine (CQ) for 48 h. Alternatively, 50 µM CQ treatment and/or starvation were applied in the last 3 h of the 48 h incubation period. Immunostaining was performed as described previously [18]. Briefly, cells were washed twice with PBS and fixed with 4% paraformaldehyde (PFA) for 10 min at room temperature. After four times washing with PBS they were permeabilized with ice cold methanol for 1 min and washed again four times with PBS. Then cells were incubated in blocking buffer (PBS containing 2 mg/mL bovine serum albumin, 1% fish gelatin, 0.1% Triton-X 100, 5% goat serum) for 1 h at RT and then immunostained with a primary antibody for 1 h at RT. After three washes with PBS, cells were incubated with secondary antibodies for 1 h at RT and washed three times with PBS. The following primary antibodies were used in this study: Mouse monoclonal anti-PMCA4b (JA3, 1:1000, cat. # MABN1801, Sigma-Aldrich), rabbit polyclonal anti-EEA1 (1:250, cat. # Ab2900, Abcam, Cambridge, UK), rabbit monoclonal antibodies (Cell Signaling Technology): anti-Rab7 (D95F2) XP (1:100, cat. # 9367), anti-Rab11 (1:100, cat. # 5589), anti-LAMP1 (D2D11)XP (1:200, cat. # 9091), anti-LC3A/B (1:100, cat. # 12741). As a secondary antibody, AlexaFlour-594 conjugated anti-rabbit IgG (Invitrogen, Waltham, MA, USA) and AlexaFlour-488 conjugated anti-mouse IgG (Invitrogen) were used. Images were taken by a LSM710, confocal laser scanning microscope (Zeiss, Oberkochen, Germany) equipped with a 63× oil objective.

For the experiments with Hela-GFP-PMCA4b cells, the cells were seeded in 8-well Lab-Tek II chambered cover-glass (Nunc) and allowed to adhere overnight. Next day, cells were treated with 10 µM p38 inhibitor for 48 h and 2.6 µg/mL anisomycin was added for the last 1 h of the 48 h incubation time. Cells were fixed with 4% paraformaldehyde (PFA) for 10 min at room temperature followed by washing with PBS. Images were taken by a confocal laser scanning microscope (Zeiss, LSM800, 40× objective).

HEK-mCherry-MKK6EE-Dox cells were seeded in an 8-well Lab-Tek II chambered coverglass (Nunc) and allowed to adhere overnight. The following day cells were transfected with SB-CAG-GFP-PMCA4b-CAG-Puro, as described in the previous section. Twenty four hours post transfection the medium was changed and 10 µM p38 inhibitor was added to the cells for an additional 25 h. Then, 2 µg/mL doxycycline was added either alone or in combination with the p38 inhibitor for 24 h. Images were taken by a confocal laser scanning microscope (Zeiss, LSM800, 40× objective).

#### 2.2.4. Ca^2+^ Signal Measurements

A375-GCAMP6 cells were seeded in an 8-well Lab-Tek II chambered coverglass (Nunc) and allowed to adhere overnight. The following day cells were treated with 0.5 µM vemurafenib, 10 µM P38 inhibitor (SB202190) and DMSO as a vehicle control. After 48 h incubation, cells were washed two times with HBSS buffer (20 mM HEPES, pH7.4, 2 mM CaCl_2_, 0.9 mM MgCl_2_ and 10% FBS). Calcium influx was initiated by the addition of 2 μM calcium ionophore A23187 (Sigma-Aldrich). Images were taken every 0.3 s by an IX-81 confocal laser scanning microscope (Olympus, Hamburg, Germany) equipped with a 60×, oil immersion objective and Fluoview FV500 software v4.1. Z-resolution was set to 1 µm. The relative fluorescence intensities were calculated as F/F0 (where F0 was the average initial fluorescence). Data were analyzed with the GraphPad Prism software v5.01 (GraphPad Software Inc., La Jolla, CA, USA).

#### 2.2.5. Transmission Electron Microscopy (TEM)

Cells were cultured, treated with CQ for 3 h, harvested, washed with DMEM-free serum and then fixed with 0.2 M Na-Cacodylate, 16% formaldehyde, 8% glutaraldehyde, 29.2 mM sucrose and 1 M CaCl_2_ for 1 h. After washing with 0.1 M cacodylate buffer, 1% uranyl-acetate was used for contrasting followed by embedding the samples into LR Gold resin. After ultrathin sectioning the samples were treated with 5% H_2_O_2_ for 5 min. After washing again, the samples were treated with 0.3% Na-borohydride, 50 mM glycine, 50 mM NH4Cl and 0.05 M TBS followed by washing and blocking in 3% milk for 30 min. The samples were immunostained with anti-GFP antibody (chicken, 1:100) (A10262, Invitrogen) overnight and then incubated with the secondary antibody 12 nm anti-Chicken gold (1:40) (703-205-155, Jackson) for 4 h. Pb-citrate was used for contrast staining. Images were taken by JEM-1011 transmission electron microscope (Jeol) equipped with a Morada digital camera (Olympus) using iTEM software (Olympus).

#### 2.2.6. Sulforhodamine B (SRB) Assay

A375 and A375-GFP-4b melanoma cells were seeded in a 96-well plate at 5000 cells/well and cultured overnight. The following day, cells were treated with p38 inhibitor or vemurafenib at the indicated concentrations and incubated for 48 h. Cells were fixed with 10% (*w*/*v*) trichloroacetic acid at 4°C for 1 h, washed with water, left to dry and then stained with 0.4% (*w*/*v*) SRB for 15 min at room temperature. The excess stain was washed with 1% (*v*/*v*) acetic acid solution and the bound SRB dye was solubilized in 10 mM Tris base solution for 10 min at room temperature with agitation. O.D was measured at 570 nm using a microplate reader (EL800, BioTek Instruments, Winooski, VT, USA).

#### 2.2.7. Cell Cycle Analysis

A375, A375-GFP-PMCA4b and MEWO cells were seeded in a 6-well plate and after overnight attachment the cells were treated with 10 μM p38 inhibitor (SB202190), 0.5 μM vemurafenib for 48 h. Cell cycle analysis was done as described previously [22]. The ratio of cells in each sub-phase was determined based on the DNA content. Briefly, cells were trypsinized and lysed with lysis buffer then stained with DAPI for 5 min at 37 °C. A stabilization buffer was added and 10 μl of each sample was loaded on 8-well NC slide. The NucleoCounter NC-3000™ system (Chemometec, Lillerød, Denmark) was used to quantify fluorescence.

#### 2.2.8. Colony-Forming Assay

A375 and A375-GFP-PMCA4b cells were seeded into a 6-well plate. The following day, A375 cells were treated with 0.5 µM vemurafenib, 10 µM p38 inhibitor (SB202190) and incubated for an additional 7 days. Media were changed and treatments refreshed on the fourth day. The colonies formed were fixed with (3:1) methanol:acetic acid for 30 min, washed with PBS and then stained with 0.5% crystal violet for 30 min. The crystal violet was then solubilized by 2% SDS with shaking. The O.D was measured at 570 nm using microplate reader (EL800, BioTek Instruments).

#### 2.2.9. siRNA Transfection

A375 or A375-GFP-PMCA4b melanoma cells were seeded into 6-well plates (1.3 × 10^5^/well) and allowed to adhere overnight. Cells were transiently transfected with siRNA using DharmaFECT 1 transfection reagent (Dharmacon Research Inc., Cambridge, UK) according to the manufacturer instructions. The following siRNA treatments were used: ON-Target plus SMARTpool PMCA4b (ATP2B4) siRNA (50 nM, cat. # L-006118-00-005, Dharmacon Research, Inc.), SignalSilence^®^ p38 MAPK siRNA I (100 nM, cat. #65645, Cell Signalling Technology), SignalSilence^®^ control siRNA (50nM, cat. #65685, Cell Signalling Technology). After 24-h transfection, the medium was changed and 10 µM p38 inhibitor was added for an additional 48 h. The total incubation time for siRNA transfection was 72 h. Protein expression from total cell lysates (30 µg protein per sample) was analyzed by western blot with anti-PMCA4 (JA9) antibody. Anti-β-tubulin antibody was used as a loading control. Densitometric analysis of the western blots was done using the ImageJ software v1.42q.

### 2.3. In vitro Cell Function Assays

#### 2.3.1. Cell Migration Assay

Directional migration of cells—transfected and treated as described in the previous paragraph—was assessed using the modified Boyden chamber assay [22]. The cells were harvested and seeded in the upper chamber of a 48-well Boyden chamber (Neuro probe, Gaithersburg, MD, USA) while in the lower chamber fibronectin was added as attractant (100 μg/mL in serum free media, Millipore). In between the two chambers a 10 μm-thick uncoated Nucleopore membrane (Whatman) with a pore diameter of 8 μm was placed. The cells were incubated for 3 h at 37 °C to allow them to migrate. Then the membrane was removed and cells on the upper side of the membrane were scrapped while cells on the lower side fixed with methanol and stained with Toluidine blue. Images were taken using a light microscope, 10x objective lens. The number of migrated cells/field were counted.

#### 2.3.2. Spheroid-Forming Assay

For a spheroid to form, a total of 150 A375 and A375-GFP-PMCA4b melanoma cells were seeded onto a rounded bottom 96-well plate coated with 5 mg/mL poly-HEMA (2-Hydroxyethyl methacrylate, Sigma-Aldrich) and incubated at 37ºC in a 5% CO_2_ incubator for three days. On the third day cells were treated with 0.5 µM vemurafenib or 10 µM P38 inhibitor (SB202190) and spheroids incubated for an additional 6 days. Spheroid formation was detected and photographed using microscopy at day 0, 3 and 6. The spheroid area and radius were analyzed using the ImageJ software v1.42q and spheroid volume was calculated as described previously [25].

For confocal microscopy A375-GFP-PMCA4b spheroids with and without treatments were transferred to an 8-well Lab-Tek II chambered coverglass (Nunc) then fixed with 4% PFA for 15 min, washed three times with PBS and then covered with VECTASHIED anti-fade mounting media (Vector Laboratories, Burlingame, CA, USA). A Z-stack images were taken using a Zeiss ApoTome microscope, 10× objective.

#### 2.3.3. Reversal of Multicellular Spheroid (MCS) Formation

This protocol was done as described previously [26] with some modifications. Spheroids were formed from A375 and A375-GFP-4b cells similar to that described in the previous section. On the fourth day, to induce reversal of spheroids, MSCs were transferred to a regular 24-well plate that allowed adhesion and reattachment of cells. After an overnight attachment, 10 µM p38 inhibitor (SB202190) or 0.5 µM vemurafenib were added to the media, as indicated. Cells were allowed to migrate from the spheroid into the surface of the plate for an additional 48 h. The cells were fixed with a 3:1 methanol:acetic acid for 30 min, washed with PBS and then stained with 0.5% crystal violet for 30 min. Images were taken for the whole plate and the surface area of each migrated MCS was measured by ImageJ software v1.42q. For confocal microscopy studies cells were fixed, permeabilized with 0.1% triton X-100 (Sigma-Aldrich) and stained with TRITC-Phalloidin (Sigma-Aldrich) (0.1 μg/mL) and DAPI (1 µM). Images were taken by confocal laser scanning microscopes (Zeiss, LSM800, 40× objective).

### 2.4. Statistical Analysis

Unless indicated otherwise, all data were presented as the mean ± standard deviation (SD) from three independent experiments. GraphPad Prism software (v5.01) was used to perform statistical analyses. The differences between the control and the experimental groups were determined by one-way analysis of variance (ANOVA) and post-hoc tests were done using Dunnett’s multiple comparison test to determine the levels of significance. For comparison of two groups, a T-test was used. For the spheroid formation experiment, where different doses and different days were used, a two-way ANOVA was used followed with post-hoc test (Bonferroni test). The levels of significance were: ^*^p  <  0.05, ^**^p  <  0.005 and ^***^p  <  0.001. In all cases, *p*  <  0.05 was considered significant.

## 3. Results

### 3.1. Inhibition of p38 MAPK—But Not JNK or NF-kB—Upregulates PMCA4b in BRAF Mutant Melanoma Cells

**Figure 1:**
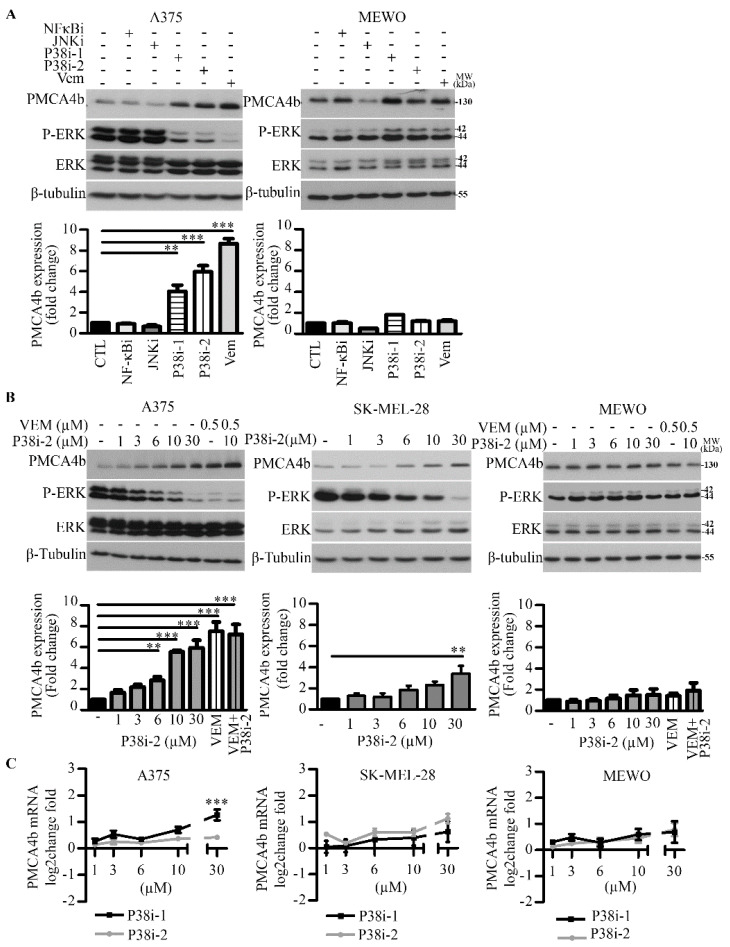

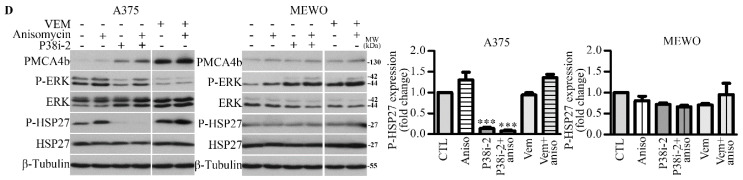
In BRAF mutant melanoma cells inhibition of p38 MAPK activity resulted in increased PMCA4b protein level while inhibition of the JNK and NF-kB pathways had no effect. (**A**) BRAF mutant (A375) and BRAF wild type cells (MEWO) were treated with inhibitors for NF-kB (BAY11-7082), JNK (SP600125), p38i-1 (SB203580) and p38i-2 (SB202190) at 10 µM concentrations for 48 h. 0.5 µM BRAF inhibitor (vemurafenib) was used as a positive control for PMCA4b protein expression. DMSO treatment was included as a vehicle control. (**B**) A375, SK-MEL-28 and MEWO cells were treated with increasing concentrations of the p38i-2 inhibitor for 48 h. In A375 and MEWO cells a combination of vemurafenib (0.5 µM) and p38i-2 inhibitor (10 µM) was tested after 48 h treatment by Western blotting. (**A**,**B**) β-tubulin was used as a loading control. Bars represent means ± SE from three independent experiments. (**C**) A375, SK-MEL-28 and MEWO cells were treated with p38i-1 SB203580 and p38i-2 inhibitor SB202190 at increasing doses for 48 h. Total mRNA was extracted and the level of PMCA4b mRNA was analyzed using qPCR. Data represent means ± SE from three independent experiments. (**D**) A375 and MEWO cells were treated with 0.5 µM vemurafenib, 10 µM p38i-2 SB202190, 2.6 µg/mL anisomycin (positive activator of p38) alone or in combination with 10 µM SB202190 for 48 h. Protein expression from total cell lysates was analyzed by Western. β-tubulin was used as a loading control. Bars represent means ± SE from three (A375) and two (MEWO) independent experiments.

In our previous study, we found that PMCA4b expression was downregulated in BRAF mutant melanoma cell lines and inhibition of the Ras-BRAF-MEK-ERK pathway markedly increased it at both the mRNA and protein levels [17]. In this study, we examined the role of p38, JNK and NF-κB pathways in PMCA4b regulation, as all have been found activated in BRAF mutant melanomas [4,5,6]. We treated BRAF mutant (A375 and SK-MEL-28) and BRAF wild type MEWO melanoma cells with specific inhibitors of JNK (SP600125), NF-κB (BAY11-7082) and p38 (SB203580 and SB202190). We found that inhibition of the p38 MAPK pathway strongly enhanced PMCA4b protein level and this effect was specific to the BRAF mutant cells. In contrast to the p38 inhibitors, the JNK and NF-κB inhibitors did not influence the protein level of PMCA4b (Figure 1A and Figure S1). We observed down-regulation of P-ERK upon p38 inhibitor treatment that may indicate crosstalk between the BRAF and p38 MAPK pathways or limited specificity of the inhibitors.

In the A375 cells PMCA4b protein level was significantly increased after 48 h of treatment with the p38 SB202190 inhibitor at 10 µM concentration while in the SK-MEL-28 cells a higher dose (30 µM) was needed to reach maximum effect (Figure 1B and Figure S2A1,A2). It is worth mentioning that this effect was similar to that seen with vemurafenib and that the combination of vemurafenib and p38 inhibitors was not additive. However, in contrast to the BRAF inhibitor [17] the p38 inhibitors had no significant effect on the PMCA4b mRNA level tested at increasing p38 inhibitor concentrations (Figure 1C) and at different time points (Figure S2B). A significant increase in PMCA4b mRNA expression level could be detected only in A375 cells at a relatively high p38 inhibitor SB203580 dose (30 µM). Therefore, we assume post-translational regulation of PMCA4b by p38 MAPK that could result in increased PMCA4b steady state level independent of the BRAF/MEK/ERK pathway. We tested two different p38 inhibitors, as both had similar effects, in the following experiments we used only SB202190.

It has been suggested that p38 MAPK is activated in BRAF mutant cells, therefore, we compared the effect of the p38 inhibitor SB202190 on the p38 MAPK substrate HSP27 in A375 and MEWO cells. We found that the p38 inhibitor alone or in combination with the activator anisomycin decreased the phosphorylation status of HSP27 (P-HSP27) in the BRAF mutant cells but had no effect on that of the BRAF wild type cells (Figure 1D). In agreement with previous publications, we found that p38 MAPK was active in the BRAF mutant A375 cells [4,27].

### 3.2. Inhibition of p38 MAPK Increased Stability and Plasma Membrane Abundance of PMCA4b Resulting in Enhanced Ca^2+^ Clearance

**Figure 2:**
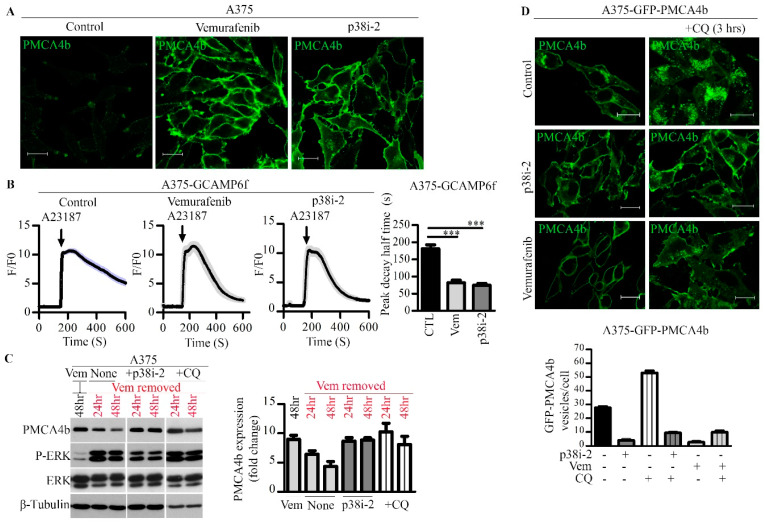
Inhibition of p38 augmented PMCA4b abundance in the plasma membrane protecting it from degradation and resulting in increased Ca^2+^ clearance. In contrast, chloroquine (CQ) trapped the protein in intracellular compartments. (**A**) Immunofluorescence staining of BRAF mutant cells (A375) with anti-PMCA4b antibody (JA3) after treatment with 0.5 µM vemurafenib and 10 µM p38i-2 (SB202190) for 48 h. Scale bar, 20µm. (**B**) A375-GCAMP6f cells were treated with 0.5 µM vemurafenib and 10 µM p38i-2 (SB202190) for 48 h. Intracellular Ca^2+^ signal was initiated by the addition of 2 µM A23187 (arrow), as indicated. Data represent fluorescent intensity values (F/F0) of 14 to 15 cells and are representative of two independent determinations. Half peak decay time was determined for the treated cells compared to control. Bar graphs are mean ± SE. (**C**) A375 cells were treated with vemurafanib for 48 h. At the end of the 48-h incubation period vemurafenib was removed and cells were incubated for an additional 24 and 48 h. At the time of vemurafenib removal, 10 µM p38i-2 inhibitor SB202190 or 10 µM chloroquine (CQ) was added to the media and protein level was analyzed by Western blotting. Bars represent means ± SE. (**D**) Confocal microscopy images of A375-GFP-PMCA4b cells after treatment with 10 µM p38i-2 inhibitor or 0.5 µM vemurafenib for 48 h, and 50 µM chloroquine (CQ) for 3 h alone or in combination. Scale bar, 20 µm. GFP-PMCA4b positive perinuclear vesicles/cell were counted (eight cells/group). Bars represent means ± SE.

Confocal microscopy analysis showed enhanced PMCA4b abundance in the plasma membrane of A375 cells in response to the p38 inhibitor SB202190, and this effect was similar to that seen in the vemurafenib treated cells (Figure 2A). To study if this increase in PMCA4b abundance is reflected in an enhanced Ca^2+^ removing capacity of the cells, we performed Ca^2+^ signaling measurements using A375 cells stably expressing a genetically encoded green fluorescent Ca^2+^ sensor GCAMP6f [23] and confocal imaging. Ca^2+^ signaling was initiated by the addition of the Ca^2+^ ionophore A23187 (2 µM) that allows Ca^2+^ to enter the cells independent of the Ca^2+^ channels. As shown in Figure 2B, Ca^2+^ was cleared much faster (T_1/2_ = 74.6 ± s) from the cytosol of the p38 inhibitor treated cells than from the control cells (T_1/2_ = 181.1 ± s). This effect was similar to that seen in the vemurafenib-treated cells (T_1/2_ = 81.8 ± s).

Our results suggested that p38 MAPK regulated PMCA4b abundance at the protein level but not at the mRNA level. Therefore, we hypothesized that p38 MAPK affected the stability of PMCA4b in the BRAF mutant A375 cells. To test our hypothesis, we treated the cells first with the BRAF inhibitor vemurafenib to increase PMCA4b expression. After vemurafenib removal, the PMCA4b protein level declined near to its low basal level within the 48-h post-treatment period (Figure 2C and Figure S3). The addition of the p38 inhibitor SB202190 or the lysosomal inhibitor chloroquine (CQ) blocked the decay effectively. After the removal of vemurafenib the cells restore their P-ERK levels as expected that was reduced again by the p38 inhibitor, however, less effectively than when added without pre-treatment suggesting alterations in the crosstalk between these pathways.

To understand how the p38 inhibitor and CQ helped stabilizing the pump, we studied subcellular localization of GFP-tagged PMCA4b by confocal imaging in a GFP-PMCA4b expressing A375 stable cell line. A relatively strong GFP-PMCA4b fluorescence signal was associated with vesicles at the perinuclear region in the control cells that was further enhanced with CQ treatment (Figure 2D) suggesting that CQ inhibited PMCA4b degradation at the lysosomal level. Treatment of the cells with the p38 inhibitor with or without CQ abolished the perinuclear GFP-PMCA4b signal nearly completely while the abundance of PMCA increased in the plasma membrane. These results suggested that p38 MAPK activation destabilized the pump by inducing its internalization, and inhibition of p38 could reverse this effect. It is important to note that vemurafenib also reduced the perinuclear GFP-PMCA4b signal even in the presence of CQ suggesting that activation of the RAF/MEK/ERK pathway also can affect PMCA4b stability that might explain the lack of additivity of the BRAF and p38 inhibitors.

### 3.3. PMCA4b Is Degraded through the Endo/Lysosomal System In a p38 MAPK Dependent Manner

**Figure 3:**
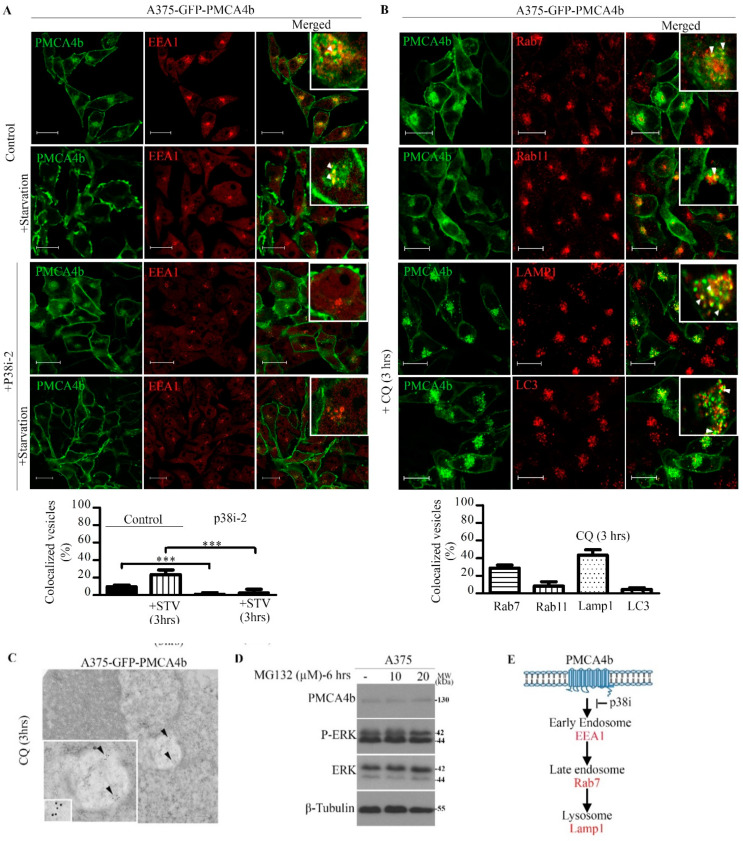
P38 inhibitor abolished GFP-PMCA4b internalization in A375 cells, and chloroquine (CQ) trapped the pump in LAMP1 positive intracellular organelles. (**A**) Confocal microscopy images of A375-GFP-PMCA4b cells after treatment with 10 µM p38i-2 inhibitor (SB202190) for 48 h with or without starvation (3 h). The cells were immunostained for the early endosomal marker EEA1. EEA1 and GFP-PMCA4b positive (yellow) vesicles were counted (six cells/group) and expressed as % of total number of GFP-PMCA4b positive vesicles. Scale bar, 20 µm. Bars represent means ± SE. (**B**) Confocal images of A375-GFP-PMCA4b immunostained with the endo-lysosomal markers Rab7, Rab11 and LAMP1, and the autophagy marker LC3 after treatment with CQ for 3 h. The number of GFP-PMCA4b vesicles that co-localize with the endo-lysosomal or autophagy markers were counted and expressed as a % of total number of PMCA4b positive vesicles. Seven to nine cells were counted in each group. Scale bar, 20 µm. Bars represent means ± SE. (**A**,**B**) Insets show higher magnification view, Scale bar, 10 µM. Arrowheads show co-localization of GFP-PMCA4b with endosomal markers. (**C**) A375-GFP-PMCA4b cells were cultured and treated with CQ for 3 h. Cells were fixed and immunostained with anti-GFP antibody (1:100) (Invitrogen, 1972783). Images were taken by JEM-1011 transmission electron microscope (Jeol). Arrowheads show positive PMCA4b protein (**D**) A375 cells were treated with the proteasome inhibitor MG132 for 6 h at the concentrations indicated. Protein expression was analyzed by Western blotting. The experiment was repeated three times. (**E**) Schematic diagram shows the pathway of PMCA4b degradation through the endo-lysosomal system.

To study further the degradation pathway of PMCA4b in BRAF mutant melanoma cells we examined its distribution between the different endocytic compartments using specific markers of the endo/lysosomal system. We used antibodies against the early endosomal marker early endosomal antigen 1 (EEA1), the late endosomal marker Ras related protein-7 (Rab7), the lysosomal marker lysosomal-associated membrane protein 1 (LAMP1), the recycling endosomal marker Ras related protein-11 (Rab11) and the autophagosome marker microtubule-associated protein 1A/1B-light chain 3 (LC3) (Figure 3). We found that 9.3% of the GFP-PMCA4b vesicles were EEA1 positive that increased to 23.5% after 3 h of serum starvation (Figure 3A). Inhibition of p38 blocked the formation of the GFP-PMCA4b-EEA1 vesicles nearly completely with or without starvation suggesting that p38 MAPK modulates PMCA4b degradation at the early endosomal level by inducing its internalization. Analyzing further the trafficking of GFP-PMCA4b along the endocytic compartments in untreated cells we found 28.8% Rab7, 8.3% Rab11, 4.6% LC3 and 43.4% LAMP1 positive GFP-PMCA4b vesicles confirming that at the end of the journey PMCA4b is degraded in the lysosomes (Figure 3B). Lysosomal degradation of the pump was also confirmed by transmission electron microscopy showing GFP-PMCA4b positive puncta within the autolysosome in A375-GFP-PMCA4b cells (Figure 3C). Importantly, the proteasome inhibitor MG132 did not have any effect on PMCA4b indicating that the proteasomal system does not participate in the degradation of the pump in these cells (Figure 3D). In summary, we show for the first time that p38 MAPK -inhibition reduced internalization of PMCA4b, and hence its degradation through the endo-lysosomal pathway. (Figure 3E).

### 3.4. P38 MAPK is a Modulator of PMCA4b Trafficking

**Figure 4:**
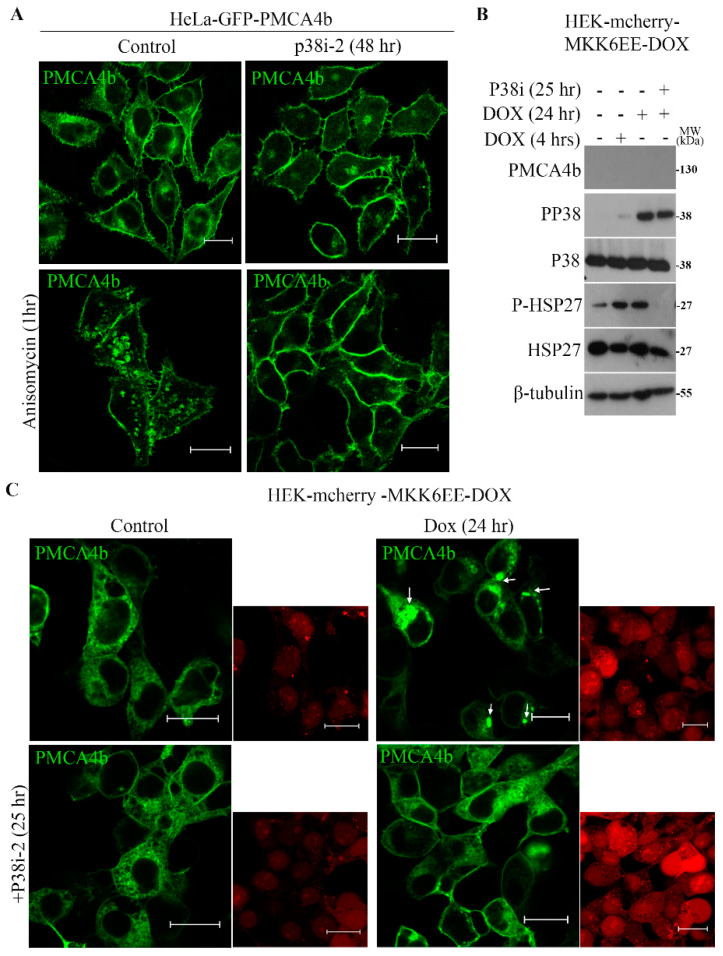
Activation of P38 MAPK induced GFP-PMCA4b internalization in the BRAF wild type HeLa cells and in a non-tumorigenic doxycycline inducible HEK-mCherry-MKK6 cell model system. (**A**) Confocal microscopy images of Hela-cells stably expressing GFP-PMCA4b treated with 10 µM of p38i-2 inhibitor SB202190 for 48 h or 2.7 µg/mL anisomycin for 1 h, and in combination. Scale bar, 20 µm. (**B**) HEK-mCherry-MKK6EE-Dox inducible cells were seeded in 6 well plate and after 24 h 10 µM p38i-2 inhibitor SB202190 was added and incubated for a total of 25 h. During the p38i-2 inhibitor treatment 2 µg/mL doxycycline was added to the media so that MKK6 was induced for 4 or 24 h, respectively. Protein expression was analyzed by western blotting. (**C**) Confocal microscopy images of HEK-mCherry-MKK6EE-Dox cells transiently transfected with the SB-CAG-GFP-PMCA4b-CAG-Puro plasmid DNA construct for 24 h. After transfection, the cells were incubated with 10 µM of p38i-2 for 25 h. To induce MKK6, 2 µg/mL doxycycline was added 1 h after p38i-2 and incubation was followed for an additional 24 h. Arrowheads show GFP-PMCA4b positive vesicles, the images on the side show mCherry signal for the same cells, Scale bar, 20 µm.

To confirm that p38 MAPK is a key controller of PMCA4b plasma membrane protein level and internalization, we used additional human cell models of different origin. Since the cervical cancer cells HeLa have low basal p38 MAPK activity [28] we generated a GFP-PMCA4b expressing HeLa cell line, in which p38 activity was induced by anisomycin. After 1-h treatment, anisomycin strongly increased the number of GFP-PMCA4b positive vesicles inside the cells that was blocked nearly completely with the p38 inhibitor, while the p38 inhibitor alone did not have any effect (Figure 4A).

To prove that this effect was specific to p38 MAPK, we used the human embryonic kidney (HEK) cell line HEK-mCherry-MKK6-Dox, where p38 activation could be induced by doxycycline. The Western blot in Figure 4B shows that doxycycline increased the active form of p38 MAPK (pp38) and its substrate P-HSP27 within 24 h and that this effect was fully prevented by the p38-specific inhibitor SB202190. Then, we transiently transfected HEK-mCherry-MKK6-Dox cells with GFP-PMCA4b and determined localization of the GFP-tagged pump by confocal microscopy 24 h after transfection. The images in Figure 4C show clearly that treatment of the cells with doxycycline induced internalization of GFP-PMCA4b into vesicle-like intracellular compartments that was prevented by p38 inhibitor treatment. The inhibitor alone did not affect the distribution of the GFP-PMCA4b signal relative to the control cells. In summary, these data confirmed that activation of p38 MAPK was required for the enhanced PMCA4b trafficking and highlights the role of changes in p38 MAPK activity in modulating PMCA4b stability in different cell types.

### 3.5. The P38 Inhibitor Induced Cell Cycle Arrest and Reduced Colony Formation but Displayed Low Cytotoxicity

**Figure 5:**
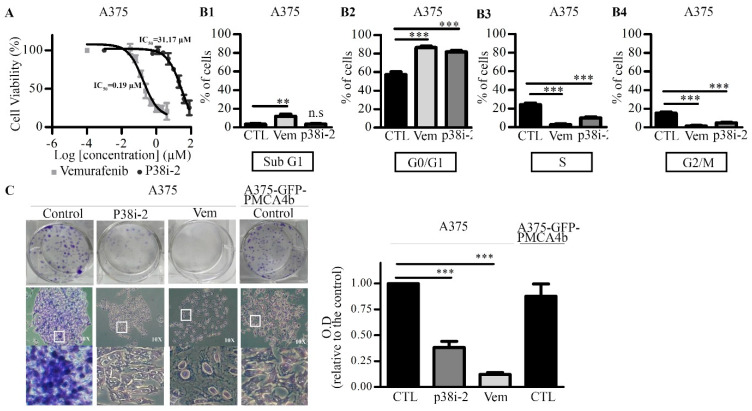
P38 inhibitor reduced proliferation and colony formation of A375 cells. (**A**) A375 cells were treated with vemurafenib or SB202190 inhibitors at increasing concentrations. After a 48-h incubation period cell viability was assessed by the SRB assay. (**B1**–**4**) Cell cycle analysis was performed after 48-h 0.5 µM vemurafenib or 10 µM p38i-2 inhibitor (SB202190) treatments. Data are means ± SE of three independent experiments. (**C**) A375 and A375-GFP-PMCA4b cells were treated with 0.5 μM vemurafenib or 10 μM SB202190 for 7 days (media and treatments were changed on the fourth day). Cells were fixed and stained with 0.5% crystal violet. Data are means ± SD of three independent experiments.

Using the SRB assay, we found that the p38 inhibitor reduced cell viability at relatively high concentrations with no significant differences between the A375 and the A375-GFP-PMCA4b cells (Figure 5A and Figure S4A). At the effective p38 inhibitor dose (10 µM) used in our experiments 75.3% of the cells were viable. P38 inhibitor treatment also decreased cell proliferation by decreasing the number of cells in the synthesis S and the G2/M phase, resulting in an increased number of cells in the resting Go/G1 phase without affecting cell toxicity, as indicated by the lack of cell accumulation in the Sub-G1 phase (Figure 5B1–B4). PMCA4b did not contribute to any of these effects (Figure S4B) in good correlation with our previous findings indicating that PMCA4b over-expression does not affect cell proliferation tested by the BrdU incorporation assay [17]. Neither the p38 inhibitor nor vemurafenib affected any of the above parameters in the BRAF wild type MEWO cells (Figure S4C,D).

In good accordance with these findings, the p38 inhibitor and vemurafenib significantly decreased colony formation of both A375 and A375-GFP-PMCA4b cells (Figure 5C). Interestingly, the number of colonies formed by the A375-GFP-PMCA4b cells was similar to that seen with the wild type cells, however, the colony morphology was different. A375 cells grew over each other to form cell aggregates, while the A375-GFP-PMCA4b cells spread over the surface and formed monolayer-type colonies. Importantly, colonies formed by the p38 inhibitor-treated A375 cells also had monolayer appearance similar to that seen with the PMCA4b over-expressing cells.

### 3.6. P38 Inhibitor Decreased Migratory Activity of A375 Melanoma Cells through PMCA4b

**Figure 6:**
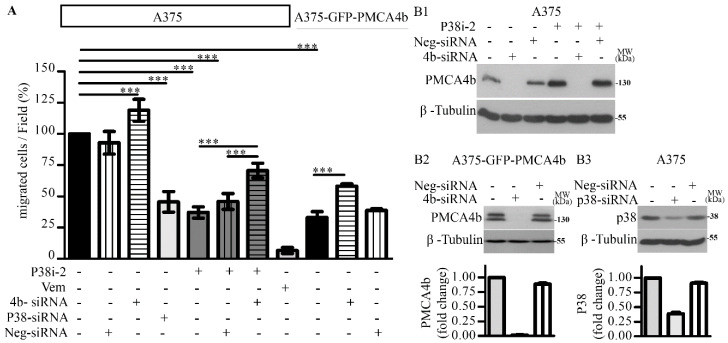
P38 inhibitor decreased cell migration at least partly through PMCA4b. (**A**) A375 and A375-GFP-PMCA4b cells were treated with 10 µM SB20290 and 0.5 µM vemurafenib for 48 h, with or without the siRNA reagents (see below). After treatments cells were harvested, counted and seeded in the upper Boyden chamber. Fibronectin was used as an attractant in the lower chamber. After 3 h incubation migrated cells were fixed and stained using Toluidine blue. Images (*n* = 4) were taken as representative fields of view from the bottom of the membrane. The bar graphs indicate the average number of migrated cells per field. Data are means ± SD of two independent experiments. (**B1**) A375 cells were transfected with PMCA4b or control siRNAs and incubated for 72 h. SB202190 was added alone or 24-h post-transfection, as indicated in the figure. (**B2**) A375-GFP-PMCA4b cells were transfected with PMCA4b and control siRNAs and incubated for 72 h. (**B3**) A375 cells were transfected with p38 MAPK or control siRNAs incubated for 72 h. Protein expression was analyzed by Western blotting using anti-PMCA4b antibody JA3. Western blots are representative of three independent determinations.

We demonstrated previously that PMCA4b over-expression decreased migration and metastatic activity of BRAF mutant A375 melanoma cells [17]. Therefore, we tested if the p38 inhibitor has a similar effect using the Boyden chamber-based directional cell migration assay. The p38 inhibitor SB202190 decreased A375 cell migration, as only 37.0% of the cells migrated through the membrane barrier relative to the control cells (Figure 6A and Figure S5).

To assess if the effect of the p38 inhibitor was attributed to the enhanced PMCA4b protein level we downregulated PMCA4b expression with ON-Target plus SMARTpool PMCA4b siRNA and used negative non-targeting siRNA as a control in both control and p38 inhibitor treated cells. PMCA4b siRNA was able to block PMCA4b protein expression both in the wild type and A375-GFP-PMCA4b cells, while the negative siRNA did not have any effect (Figure 6B1,B2). We demonstrated that downregulation of PMCA4b: 1/enhanced migration of the control A375 cells by 20%; 2/nearly doubled the number of migrated A375-GFP-PMCA4b cells; and most importantly 3/ reversed the effect of the p38 inhibitor on cell migration increasing the number of migrated cells after p38 inhibitor treatment by 33.6% (Figure 6A, Figure S5).

Next, we reduced p38 MAPK by siRNA treatment (Figure 6B3) and found that decreasing p38 expression by 60% inhibited cell migration, as only 45.5% of the cells migrated through the Boyden membrane when compared to the control cells (Figure 6A, Figure S5). Taken together these data provided evidence for the ability of p38 MAPK in stimulating cell migration at least partly through the downregulation of PMCA4b protein level.

### 3.7. PMCA4b and p38 Inhibitor Moderately Reduce Spheroid Growth

**Figure 7:**
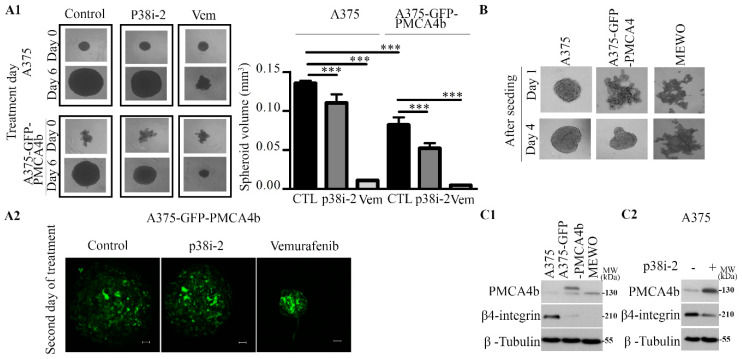
P38 inhibitor slightly reduced A375 cell spheroid growth, while A375-GFP-PMCA4b cells showed a delay in spheroid formation and MEWO cells did not form spheroids. (**A1**) A375 and A375-GFP-PMCA4b cells were seeded in POLY-HEMA treated 96 well round bottom plate and incubated for 3 days for spheroid formation. At the third day (zero-time point.), cells were treated with 0.5 μM vemurafenib or 10 μM SB202190 for 6 days. Images were taken at 0 and 6-day time points using light microscope, 4×. The spheroid area and radius were determined and spheroid volume (mm^3^) was calculated. Data are means ± SD of three independent experiments. (**A2**) For fluorescence microscopy, A375-GFP-PMCA4b cell spheroids were formed for 3 days, then 0.5 μM vemurafenib or 10 μM SB202190 were added to the media and incubated for an additional 48 h. Spheroids were fixed and Z-stack images were taken using Axio Imager.M2 microscope (ZEISS) with an ApoTome2 grid confocal unit (ZEISS), 20x objective. Scale bar, 100 µm. (**B**) A375, A375-GFP-PMCA4b and MEWO cells were seeded on POLY-HEMA treated round bottom 96-well plate and incubated for 4 days for spheroid formation. Images were taken at 1 and 4-day time points using light microscope, 4×. (**C1**) A375, A375-GFP-PMCA4b cells and MEWO cells were seeded in a 6-well plate and grown for 48 h. (**C2**) A375 cells were seeded in 6-well plate and treated with 10 μM SB202190 for 48 h. (**C1**,**C2**) Protein expression level was analyzed by Western blotting with anti-β-integrin antibody. Western blot results are representative of three independent experiments.

A three-dimensional spheroid model is considered useful to study the effect of inhibitors and drugs on cancer cell growth and proliferation [29]. Therefore, we tested how p38 inhibitor affected spheroid formation from the melanoma cells. A375 and A375-PMCA4b cells were seeded on poly-HEMA coated 96-well plates. After 3 days of spheroid formation, p38 inhibitor and vemurafenib were added at three different doses and spheroids were grown for additional 6 days (Figure S6). As shown in Figure 7A1, both p38 inhibitor and vemurafenib reduced the volume of spheroids although vemurafenib was more effective. Interestingly, A375-GFP-PMCA4b cells showed a delay in compact spheroid formation that resulted in smaller spheroids compared to the parental A375 cells by the end of the 6-day culturing period. In good accordance with the results of the present paper a substantial increase in GFP-PMCA4b protein abundance could be detected in the spheroids when A375-GFP-PMCA4b cells were treated with the p38 inhibitor (Figure 7A2). It is worth mentioning that under similar conditions the BRAF wild type MEWO cells did not form spheroids (Figure 7B).

Several proteins including integrins are involved in spheroid formation and structure [30]. Integrin β4 has been shown to modulate cell migration and cancer invasion [31], therefore, we tested the expression of integrin β4 in A375 melanoma cells. Figure 7C1 shows high integrin β4 level in the parental A375 cells that was nearly completely lost in A375-GFP-PMCA4b cells, and MEWO cells did not express this protein at all. Interestingly, the p38 inhibitor-induced increase in PMCA4b abundance coincided with decreased integrin β4 expression (Figure 7C2). These data suggest that this decrease in integrin β4 expression could be one of the factors responsible for the reduced spheroid growth and/or delay in spheroid formation in response to the p38 inhibitor and PMCA4b overexpression.

### 3.8. P38 Inhibitor Reduced Metastatic Activity of A375 Cells In Vitro

**Figure 8:**
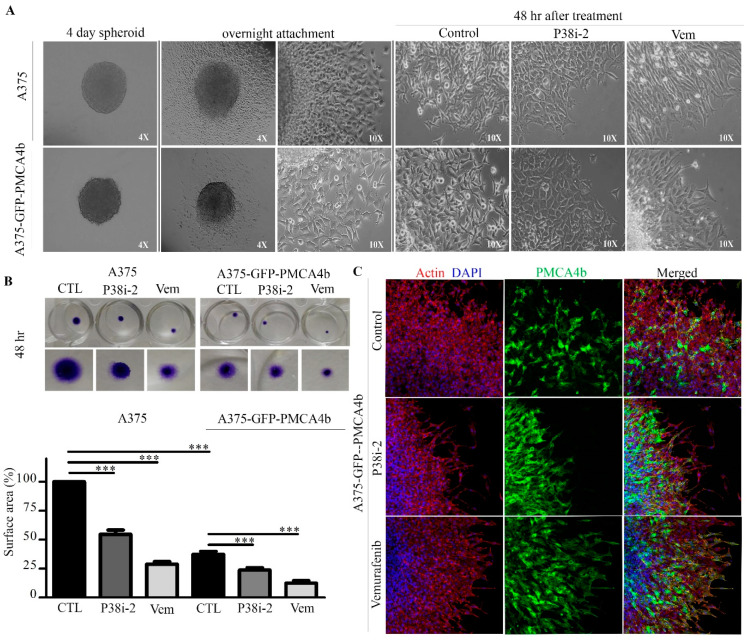
P38 inhibitor reduced metastatic activity (reversal of multicellular spheroids) of A375 cells in vitro. (**A**) A375 and A375-GFP-PMCA4b cells were seeded to form spheroids as previously described. After four days the spheroids were transferred to a 24-well plate and after overnight attachment they were treated with 0.5 μM vemurafenib or 10 μM SB202190 for 48 h. Images were taken using light microscope, 4× and 10× magnification. (**B**) Cells were fixed and stained with 0.5% crystal violet. Pictures were taken and the surface area was measured using the ImageJ software v1.42q. The surface area was calculated relative to the control. Data are means ± SD of three independent experiments. (**C**) A375-GFP-PMCA4b spheroids were generated and treated in a similar way as above. Cells were fixed and immunostained with phalloidin-TRITC and DAPI. Pictures were taken using confocal microscopy, 10×. Scale bar, 20 µm.

Reversal of anchorage-independent multicellular spheroids (MCS) into a monolayer has been suggested to test metastasis in vitro [26]. To study the metastatic activity of A375 and A375-GFP-PMCA4b cells, we cultured spheroids for 4 days and then transferred them to a 24-well plate for attachment. After 24 h when cells started to migrate p38 inhibitor or vemurafenib were added and monolayer formation was followed for additional 48 h (Figure 8A). The p38 inhibitor reduced the surface area of the metastatic MCS generated from A375 cells to 54.6% while vemurafenib to 28.9%, respectively (Figure 8B). A375-GFP-PMCA4b cells showed a substantial reduction in MCS metastasis as compared to the parental A375 cells. We also noticed that the GFP-PMCA4b signal of the cells at the spheroid outer layer was reduced and the round epithelial-like cell morphology was lost in accordance with the notion that cells with low PMCA4b migrated faster than those with high PMCA4b abundance (Figure 8C). Increasing the GFP-PMCA4b signal by inhibiting p38 and BRAF reduced the metastatic activity of the MCS, highlighting again the importance of p38 MAPK and PMCA4b in cell migration and metastasis.

## 4. Discussion

In the present study we identified the p38 MAPK pathway as a novel regulator of PMCA4b protein levels. Many p38 MAPK substrates are known, including protein kinases and transcription factors, and these substrates have various functions that are important in chromatin remodeling, protein degradation and localization, endocytosis, apoptosis, cytoskeleton dynamics and cell migration [32]. MAPK kinases (MKKs) can activate p38 MAPK upon different stimuli such as environmental stress, inflammatory cytokines and growth factors [33,34]. We showed that BRAF mutant A375 cells have high p38 MAPK activity as indicated by the high level of phosphorylation of its substrate HSP27 that was fully suppressed with the p38 inhibitor SB202190. In agreement with our findings, it has been suggested that because of the loss of the negative feedback loop between p38 and ERK both pathways could be activated simultaneously in melanoma that may contribute to the decreased p-ERK level upon p38 inhibition in BRAF mutant melanoma cells [4].

PMCA4b expression is often downregulated in cancer but the exact mechanisms involved are not fully understood [12,14]. Previously, we demonstrated that inhibition of the BRAF/MEK/ERK pathway with the BRAF specific inhibitor vemurafenib resulted in upregulation of PMCA4b at both the mRNA and protein levels in BRAF mutant cells. Here we found that inhibition of the p38 MAPK also increased PMCA4b, however, this inhibition affected PMCA4b expression only at the protein level suggesting post-translational regulation. The increased abundance of PMCA4b in the plasma membrane together with a significant decrease in its intracellular localization indicates that p38 MAPK controls stability of PMCA4b at the plasma membrane level. The mutant BRAF-specific inhibitor vemurafenib also increased plasma membrane localization of the pump suggesting that BRAF can regulate PMCA4b both at the mRNA and protein levels. Accumulation of PMCA4b in the plasma membrane resulted in enhanced Ca^2+^ clearance from the cells confirming that it remained functional after treatments.

Cells utilize the canonical endo-lysosomal degradation and autophagy pathways for membrane protein degradation when proteins are delivered to the early/late endosomal system and finally degraded by fusion with the lysosome, a highly acidic organelle with active proteases. In addition, many membrane proteins are internalized to endosomes by ubiquitination that also could act as a signal for endocytic internalization [35]. In melanoma with RAS and BRAF mutations autophagy and lysosomal functions are often deregulated to promote cell survival [36]. Many kinases, including p38 MAPK that have a role in cell proliferation and growth were found to be involved in membrane protein internalization that could lead to degradation of these proteins [28,37]. As shown in a previous study, rat brain PMCA had long half-life (12 days (±1)) with high stability and slow turnover rates [38]. In our study we demonstrate for the first time that p38 MAPK controls PMCA4b internalization using three different model systems including two tumor cell lines (BRAF mutant A375 and HeLa cells) and the doxycycline inducible non-tumorigenic HEK cell model. P38 inhibitor treatment was able to prevent p38-induced internalization of PMCA4b in all these cells. Furthermore, we demonstrated that PMCA4b is degraded through the endo-lysosomal system. Our results suggest that p38 MAPK can induce PMCA4b internalization and possibly degradation in response to stress stimuli under various physiological or pathological conditions. Previously, we identified a C-terminal di-leucine-like motif that mediates PMCA4b endocytosis [39]. Further studies are needed to test if this motif is involved in the p38 MAK induced internalization of this pump under stress conditions.

Our laboratory identified PMCA4b as a putative metastasis suppressor with no effect on cell viability. In good accordance with these findings, inhibition of p38 MAPK also decreased cell proliferation with no significant cytotoxic effect [40]. Moreover, p38 inhibitor treatment showed reduction in colony formation similarly to that reported for ovarian and colorectal cancer cell lines [41,42,43]. Interestingly, A375-GFP-PMCA4b cells also formed colonies, however, these colonies displayed monolayer clusters rather than compact cell aggregates indicating that the tumor forming ability of the PMCA4b cells was decreased. The appearance of the A375-GFP-PMCA4b cell colonies was similar to that of the p38 inhibitor treated wild type cells suggesting that PMCA4b played a role in the reduced colony formation.

The first study on the role of p38 on cell migration used endothelial cells stimulated by vascular endothelial growth factor VEGF [44]. Later, it was found that p38 regulated cell motility by inducing cytoskeletal rearrangement through the activation of the p38 substrate HSP27 [34]. In the present study, we showed that inhibition of the p38 MAPK pathway decreased A375 melanoma cell migration partly through PMCA4b. PMCAs are key regulators of intracellular Ca^2+^ concentrations maintaining basal Ca^2+^ levels and shaping the Ca^2+^ signal in an isoform dependent manner [45]. As local and global Ca^2+^ fluxes have been implicated in cell motility through the regulation of actin dynamics [46] the effect of PMCA4b could also be related to Ca^2+^ signaling. A recent study suggested that polarized distribution of store-operated Ca^2+^ channels and PMCA4 were responsible for the maintenance of the Ca^2+^ gradient needed for directional cell movement of endothelial cells [47]. Interactions between PMCA4b and actin have also been suggested to contribute to actin cytoskeleton dynamics [48]. To understand the exact mechanisms by which PMCA4b and p38 MAPK modulate cytoskeletal rearrangement, however, needs further investigation.

3D cell cultures are considered as a powerful tool for tumor growth modeling because they resemble the tumor microenvironment and organization in the human body better than 2D cell culture models [29]. In the present study we showed that p38 inhibitor treatment reduced spheroid growth in BRAF mutant cells although much less effectively than vemurafenib. Similar effects of p38 inhibitors were seen in other cancer models, as well [49,50]. Interestingly, A375-GFP-PMCA4b cells needed more time to form spheroids than the parental A375 cells and these spheroids were significantly smaller. Many factors can affect spheroid integrity and formation involving integrins. In primary and metastatic melanoma, the integrins β1 and β3 are found to be upregulated and involved in the development of melanoma metastasis [30]. Published data demonstrated that integrin β4 has a role in cell-matrix junction protein assembly [31]. In the present study we found high β4 integrin expression in A375 cells that was significantly downregulated upon PMCA4b overexpression or p38 inhibitor treatment. Because of the reduced integrin β4 expression cells may form loose integrin-ligand bonds [51] that could explain the observed delay in spheroid formation and/or metastatic activity of the cells.

A recent study [26] has developed an in vitro metastasis model characterized by the reversal of multicellular spheroids. Using this model, we found strong inhibition of A375 melanoma cell metastatic activity in response to p38 and BRAF inhibitor treatments. In good accordance with previous findings [17], A375-GFP-PMCA4b melanoma cells showed significantly reduced metastatic activity highlighting the role of PMCA4b as a metastatic suppressor.

In conclusion, this study demonstrated that p38 MAPK pathway inhibition in A375 BRAF mutant melanoma cells rescued the putative metastatic suppressor PMCA4b from degradation through the endolysosomal pathway by inhibiting its internalization from the plasma membrane and consequently increased PMCA4b stability. This effect resulted in restoration of PMCA4b function and marked reduction of migration of A375 melanoma cells nearly as efficient as inhibitors of BRAF/MEK/ERK pathway. Finding targets to prevent the degradation of metastatic suppressors could be an additional tool of counteracting resistance in metastatic cancer cells, therefore, p38 inhibitors can be considered as a putative therapeutic option for the treatment of BRAF mutant melanoma metastasis.

## Supplementary Materials

The following are available online at https://www.mdpi.com/2073-4409/9/5/1209/s1, Figure S1: P38 inhibitor upregulates PMCA4b protein level in the BRAF mutant SK-MEL-28 cell line, however, NF-kB and JNK inhibitors were not effective, Figure S2: Inhibition of p38 significantly upregulated PMCA4b after 48 h in the BRAF mutant A375 but not in the BRAF wild type MEWO cells, Figure S3: P38 inhibitor rescued PMCA4b from degradation after vemurafenib removal, Figure S4: A375-GFP-PMCA4b cells responded similarly to both p38i-2 and vemurafenib as the parental A375 cells. Neither of the inhibitors affected cell viability and cell cycle in the BRAF wild type MEWO cells, Figure S5. Microscopy images of the migrated cells corresponding to the graph in Figure 6A, Figure S6. P38 inhibitor showed less effect on spheroid growth than vemurafenib.
