# Supplementary material for: P38 MAPK Promotes Migration and Metastatic Activity of BRAF Mutant Melanoma Cells by Inducing Degradation of PMCA4b
Source: Cells. 2020 May 13;9(5):1209. doi: 10.3390/cells9051209 (PMC7290426; doi:10.3390/cells9051209)
Supplement: Supplementary file 1 [file cells-09-01209-s001.pdf]

Supplementary Figures

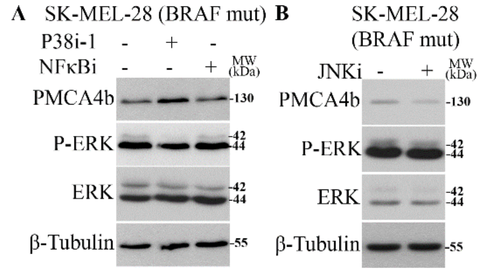

Figure S1. P38 inhibitor upregulates PMCA4b expression in the BRAF mutant SK-MEL-28 cell line, however, NF-kB and JNK inhibitors were not effective. (**A,B**) SK-MEL-28 cells were seeded in a 6- well plate and treated with (**A**) 10 μM SB203580 (p38i-1), 10 μM Bay 11-7082(NF-kB); and (**B**) with 10 μM SP600125 (JNKi) for 48 hour.

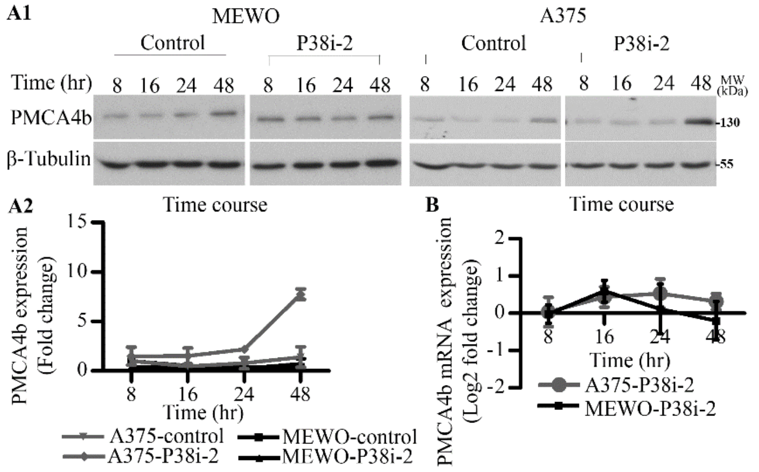

Figure S2. Inhibition of p38 significantly upregulated PMCA4b after 48 hours in the BRAF mutant A375 but not in the BRAF wild type MEWO cells. (**A1**) A375 and MEWO cell lines were treated with 10 μM p38i-2 inhibitor SB202190 for 8, 16, 24 and 48 hours. Protein expression from total cell lysates (30 μg protein per sample) was analyzed by Western blot with anti-PMCA4b antibody. β-tubulin is used as a loading control. (**A2**) Western blots were analyzed by densitometry using the ImageJ software v1.42q. Lines represent means ± SD from three independent experiments. (**B**) In a parallel experiment mRNA was extracted from each samples and analyzed using qPCR. Lines represent means ± SD from three independent experiments.

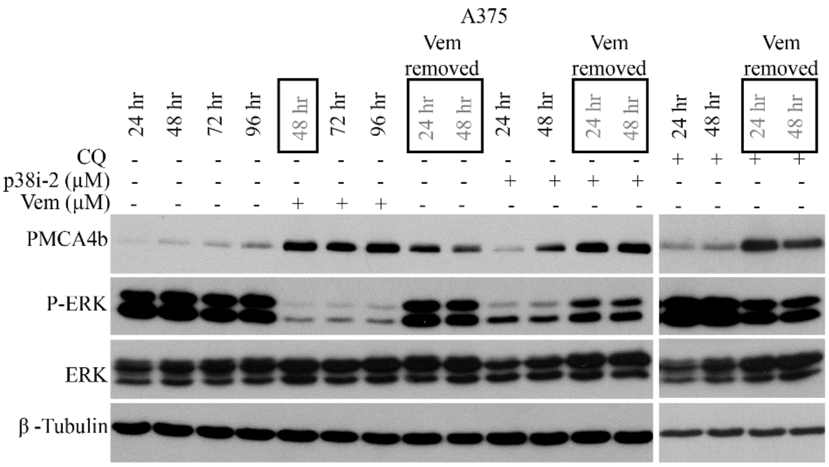

Figure S3. P38 inhibitor rescued PMCA4b from degradation after vemurafenib removal. This is the same experiment as in Fig. 2c. Briefly: A375 cells were seeded in a 6-well plate and treated with 0.5 μM vemurafanib for 48 hours. Then vemurafenib was removed and cells were incubated for additional 24 or 48 hours. During the time of vemurafenib removal, 10 μM p38 inhibitor SB202190 or 10 μM chloroquine (CQ) were added to the samples as indicated. The Western blot includes all controls that are not shown in Figure 2C for simplicity: untreated cells cultured for 24, 48, 72, 96 hours, 0.5 μM vemurafenib treatment with incubation times 48, 72 and 96 hours, 10 μM p38 inhibitor treatment with incubation times 24 and 48 hours, 10 μM CQ treatment with incubation times 24 and 48 hours. Protein expression from total cell lysates (30 μg protein per sample) was analyzed by western blot with anti-PMCA4b, anti-P-ERK and anti-ERK. β-tubulin is used as a loading control. Western blot result is representative of three independent experiment.

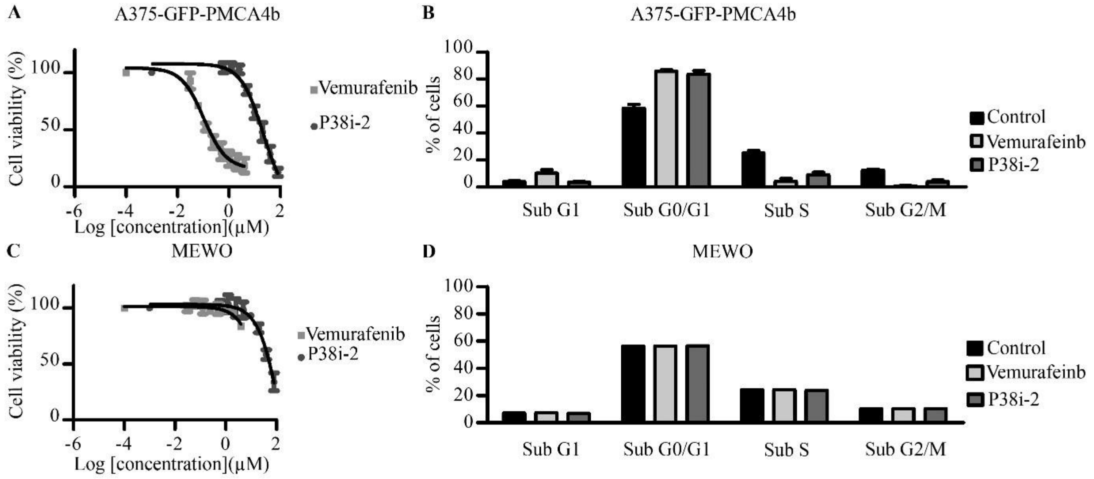

Figure S4. A375-GFP-PMCA4b cells responded similarly to both p38i-2 and vemurafenib as the parental A375 cells. Neither of the inhibitors affected cell viability and cell cycle in the BRAF wild type MEWO cells. (**A**,**C**) MEWO and A375-GFP-PMCA4b cells were seeded in a 96-well plate. After overnight attachment cells were treated with 0.5 μM vemurafenib or 10 μM p38 inhibitor SB202190 for 48 hours at eight different concentrations, as indicated. The percentage of viable cells was determined using the SRB assay. (**B**,**D**) Cell cycle analysis, the ratio of cells in each sub phase, was determined based on the DNA content. Data are means± SE of three independent experiments.

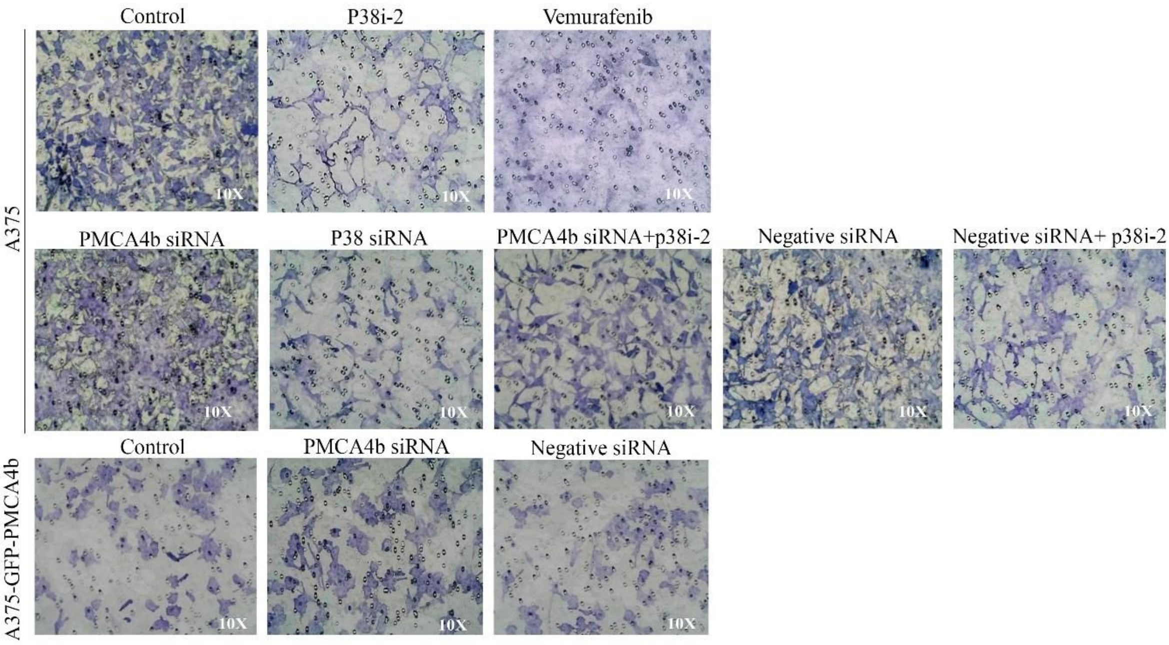

Figure S5. Microscopy images of the migrated cells corresponding to the graph in Figure 6A.

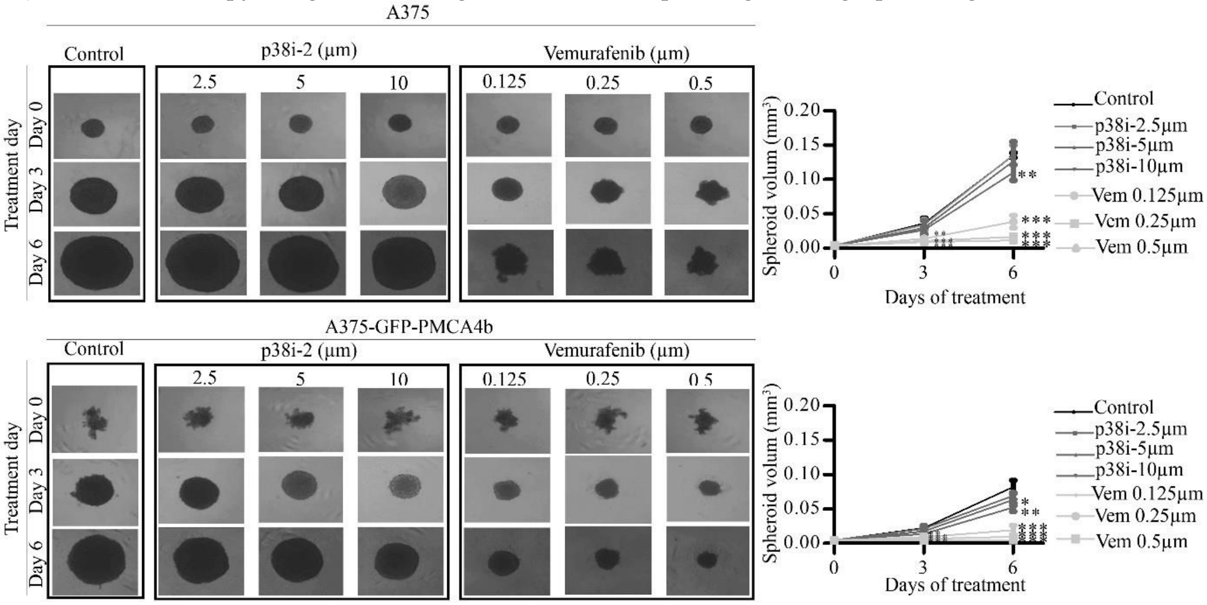

Figure S6. P38 inhibitor showed less effect on spheroid growth than vemurafenib. A375 and A375-GFP-PMCA4b cells were seeded on a POLY-HEMA treated 96-well plate with round bottom andincubated for 3 days for spheroid formation. At the third day (zero-time point.), cells were treated with 3 different doses of each inhibitor: vemurafenib (0.125, 0.25, 0.5 µM), SB202190 (2.5, 5, 10 µM) for 6 days. Images were taken at 0, 3 and 6-day time points using light microscope, 4x. The spheroid area and radius were analyzed using the ImageJ software v1.42q and spheroid volume (mm^3) was calculated. Data are means± SD of three independentexperiments.
